# Added value of 3D echocardiography in the diagnosis and prognostication of patients with right ventricular dysfunction

**DOI:** 10.3389/fcvm.2023.1263864

**Published:** 2023-12-21

**Authors:** Michael Randazzo, Francesco Maffessanti, Alekhya Kotta, Julia Grapsa, Roberto M. Lang, Karima Addetia

**Affiliations:** ^1^ Department of Medicine, Section of Cardiology, University of Chicago Heart and Vascular Center, Chicago, IL, United States; ^2^ Maria Cecilia Hospital, GVM Care & Research, Cotignola, Italy; ^3^ Department of Internal Medicine, Baylor College of Medicine, Houston, TX, United States; ^4^ Department of Cardiology, Guys and St Thomas NHS Trust, London, United Kingdom

**Keywords:** 3D echocardiography, right ventricle, prognosis, echocardiography, tricuspid valve

## Abstract

Recent inroads into percutaneous-based options for the treatment of tricuspid valve disease has brought to light how little we know about the behavior of the right ventricle in both health and disease and how incomplete our assessment of right ventricular (RV) physiology and function is using current non-invasive technology, in particular echocardiography. The purpose of this review is to provide an overview of what three-dimensional echocardiography (3DE) can offer currently to enhance RV evaluation and what the future may hold if we continue to improve the 3D evaluation of the right heart.

## Introduction

The diagnostic and prognostic importance of RV size and function is being increasingly appreciated owing both to the advent of percutaneous procedures targeted to pathologies involving the right heart and the growing interest in prognostication of various disease states that compromise the RV (1). In spite of this, reliable, non-invasive assessment of RV size and function remains elusive.

**Figure 1:**
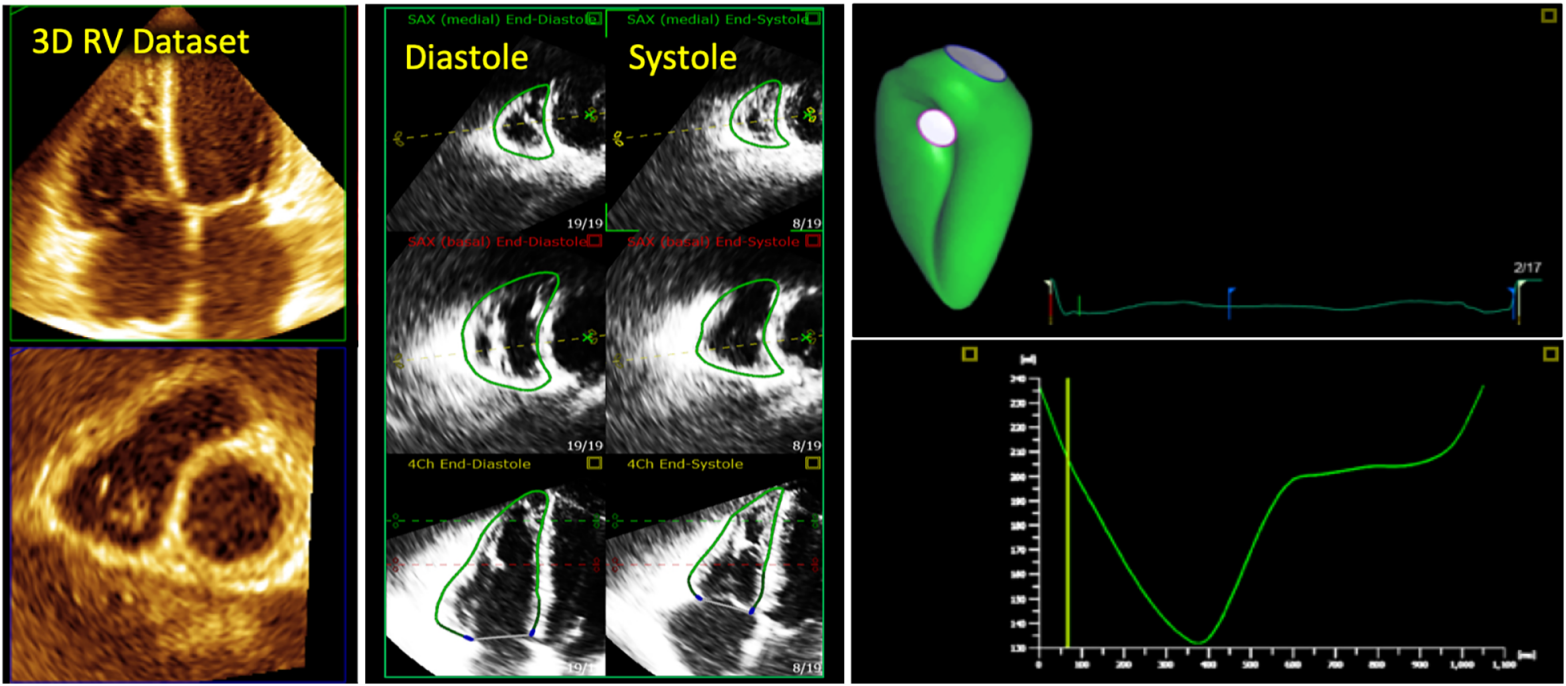
3d echocardiographic analysis of the right ventricle. Far left, 3D dataset of the right ventricle depicting the apical four-chamber and short-axis planes using multi-planar reconstruction. Middle panel shows endocardial tracings overlying the diastolic and systolic phases of the 2D short and long-axis cut-planes obtained from the 3D right ventricular dataset. Far right, top panel shows the 3D endocardial rendered surface while bottom panel shows the volumes obtained after automated software analysis throughout the cardiac cycle.

Despite being the current reference standard for RV size and function assessment, cardiovascular magnetic resonance imaging (CMR) suffers from several critical drawbacks which prevent its ubiquitous use in the clinical space. These drawbacks include, high cost, limited availability, non-portability, dependence on patient cooperation as well as numerous key contraindications and relative contraindications. Echocardiography, therefore, remains the workhorse for initial cardiac evaluation; it is cost-effective, safe, widely accessible, portable and without contraindications. It can be flexibly incorporated across multiple clinical settings. Accurate, reproducible evaluation of the RV, however, with current two-dimensional echocardiography (2DE) techniques is hindered by its anatomical location in the anterior mediastinum, irregular crescentic geometry, complex mechanics, and asymmetric remodeling. Recent advancements in 3DE have helped overcome many of these limitations by avoiding the geometrical assumptions inherent to 2D size and function assessments (2). Using 3DE, a pyramidal dataset of the RV which contains all of the structural components of the chamber including the inflow, outflow, body and apex as well as the tricuspid and pulmonary valves is acquired (Figure 1). Access to this data enables comprehensive, quantitative volumetric analysis of the RV (3). RV volume and ejection fraction (RVEF) derived from 3D datasets have been shown to strongly correlate with measurements obtained with CMR as well as adverse cardiopulmonary outcomes (4–6). These findings have recently prompted considerable exploration into the potential utility of 3DE in a vast array of clinical applications. The objective of this review is to describe 3DE acquisition and analysis methods for the RV, summarize their established diagnostic and prognostic value, and outline potential novel utilities for 3D RV imaging on the horizon and for the future.

## 2D echocardiography: analysis and limitations

**Figure 2:**
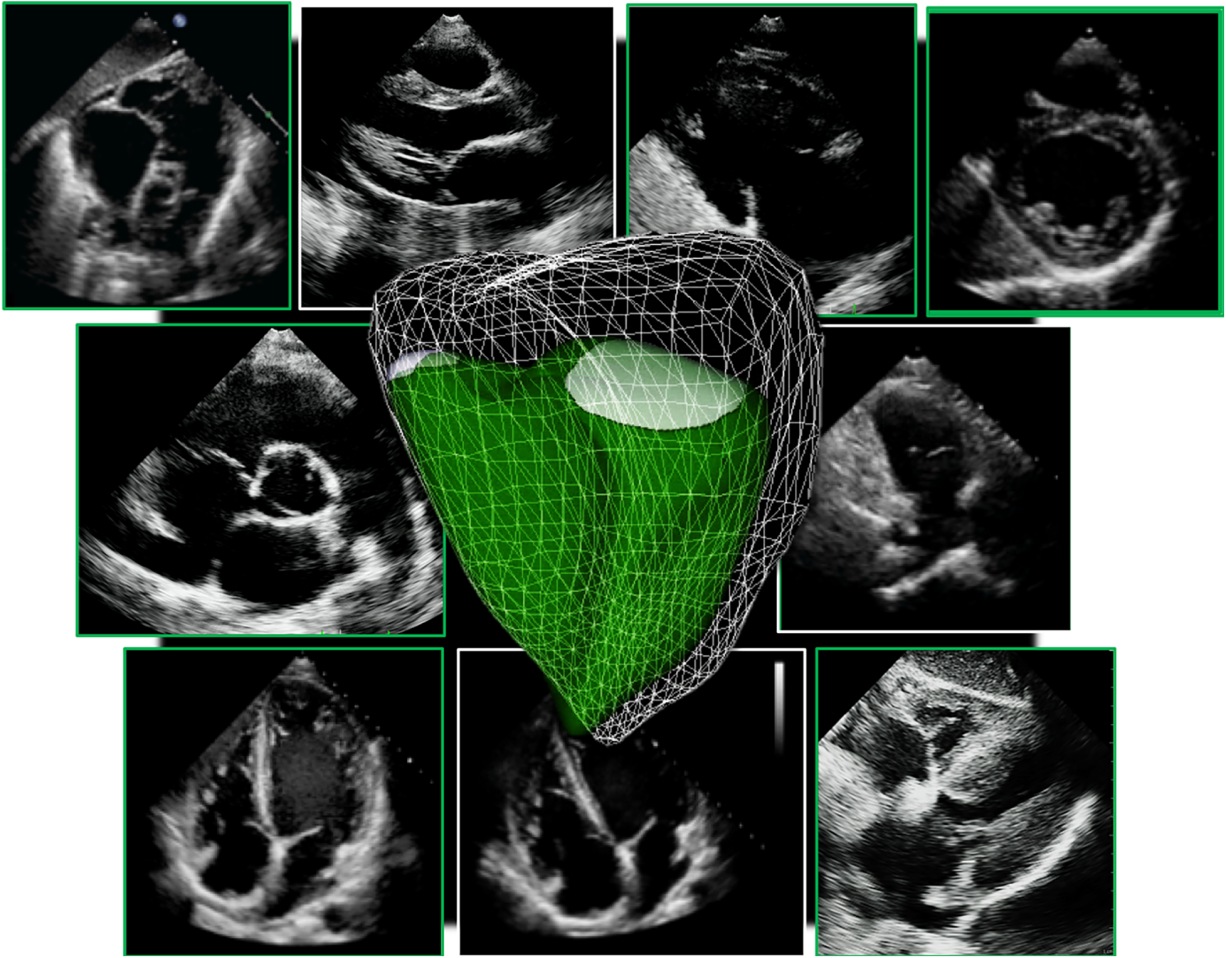
Collection of all 2D standard imaging planes used on routine transthoracic echocardiography to assess the right ventricle. A 3D right ventricle endocardial surface is superimposed on top (in green).

**Figure 3:**
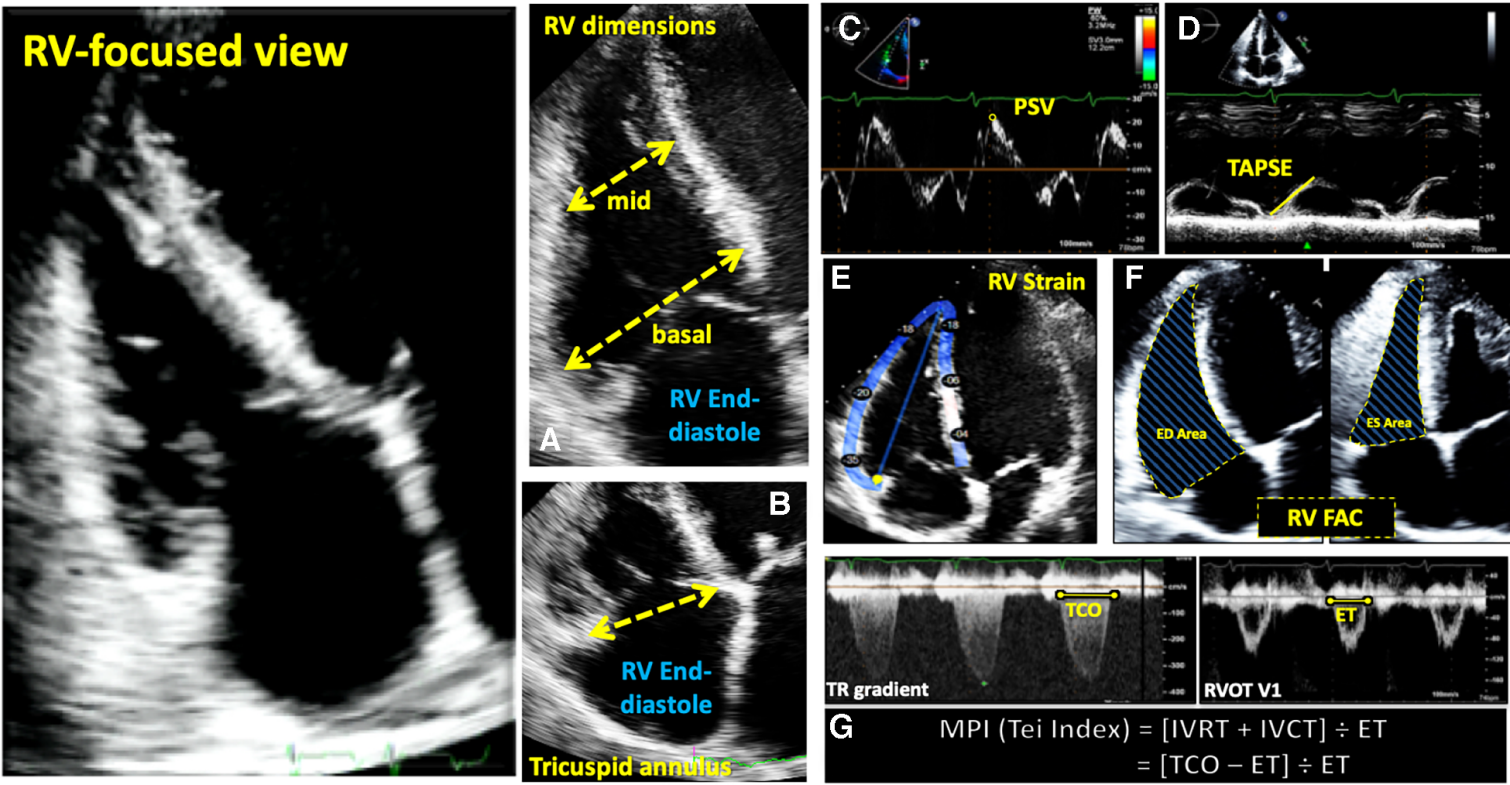
Guideline-recommended right ventricular size and function assessment. All measurements are performed in the RV focused apical 4-chamber view (far left panel). (**A**) RV mid-ventricular and basal dimensions obtained at end-diastole in the RV-focused view; (**B**) tricuspid annulus diameter obtained at end-diastole starting at the hinge-point of the non-septal TV leaflet and ending at hinge-point of the septal TV leaflet; (**C**) depiction of doppler-derived tricuspid lateral annular systolic velocity (S’) or peak systolic velocity (PSV); (**D**) m-mode is used to measure longitudinal displacement of the lateral tricuspid annulus in systole to yield tricuspid annular systolic excursion (TAPSE); (**E**) region of interest used to guide free-wall and global RV strain assessment; (**F**) RV areas obtained at end-diastole and end-systole to compute fractional area change; (**G**) Calculation of the myocardial performance index (MPI) defined as the sum of isovolumic contraction time the (IVCT) and isovolumic relaxation time (IVRT) divided by ejection time (ET) obtained from the right ventricular outflow tract initial velocity (RVOT V1). The sum of IVCT and IVRT is equal to the difference between the interval from cessation to onset of the tricuspid inflow (TCO) and ET.

2D echocardiographic evaluation of the RV requires integration of multiple imaging planes to enable optimal evaluation of RV size and function (Figure 2). Even when all views can be adequately acquired, quantitative analysis remains a regional assessment at best. RV chamber size is typically classified as normal or abnormal according to basal, mid-ventricular, and longitudinal dimensions obtained at end-diastole from the RV-focused apical 4-chamber view (Figure 3) (7). Despite this standardized approach, measurements can still vary widely based on minor differences in transducer positioning. Indeed, RV size and functional measurements have been shown to be consistently different in the RV focused view when compared with the apical 4-chamber view (8). Furthermore, volumetric estimations derived from geometric assumptions based on linear dimensions correlate poorly with volumes calculated from CMR and are discouraged (9).

Evaluation of RV systolic function on 2DE involves the integration of multiple parameters, of which the most commonly used include tricuspid annular systolic excursion (TAPSE), tissue Doppler-derived tricuspid lateral annular systolic velocity (S'), fractional area change (FAC), free-wall and four-chamber (free-wall + septal) longitudinal strain (FWS and 4CHLS respectively), and myocardial performance index (MPI) (7). Indeed, utilization of a single, global assessment of function is hindered by the complex mechanism of RV contraction that is unique from the left ventricle. In comparison to the left ventricle, the RV wall is thinner and composed of two muscular layers with longitudinally oriented myocytes in the sub-endocardium and circumferentially oriented myocytes in the sub-epicardial layer. Together, these layers contribute to RV contraction by respectively drawing the tricuspid annulus towards the apex and inwardly moving the free wall (2). Given that there is not one single accepted method for RV quantification, qualitative interpretation is often also used in clinical practice, characterizing dysfunction as mild, moderate, or severe despite poor sensitivity and notable interobserver variability (9). Routine metrics such as TAPSE and S' have demonstrated modest agreement with global RV systolic function obtained by CMR (10). These measures predominantly assess longitudinal excursion of the RV free-wall and thereby omit the contribution of other determinants of RV function. In many instances, longitudinal excursion is the most important determinant of systolic RV function. However, altered contraction mechanics and adverse remodeling in various disease states may result in under- or over-estimation of function using these methods such as in the setting of some types of pulmonary hypertension, post-cardiac surgery where systolic motion is concentrated in the transverse plane, or significant tricuspid regurgitation (TR) which produces exaggerated motion of the base (2). FAC provides a percentage estimate of global function, improving the correlation with CMR but it is highly dependent on identifying a suitable tomographic view that avoids cavity foreshortening. This limitation results in poor reproducibility. Additionally, since FAC is measured in the RV focused view, it excludes much of the RV body as well as the RV outflow tract which in itself contributes to 25%–30% of the RV volume (3, 7). Similar to FAC, FWS, a measure of myocardial shortening in the longitudinal plane, fails to incorporate the RV outflow tract and focuses on longitudinal deformation. Unlike TAPSE and RV S', FWS incorporates the entire RV free wall (or free and septal walls in the case of 4CHLS), and therefore correlates better with CMR measurements than either TAPSE or RV S' (11). Finally, MPI, or Tei index, defined as the sum of RV iso-volumetric contraction and relaxation times divided by the RV ejection time, has the ability to account for both systolic and diastolic components of RV function; however, situations such as tachycardia, elevated right atrial pressures, atrial fibrillation, and conduction system disorders prevent its consistent use (2). Overall, conventional 2D parameters (Figure 3) necessitate leveraging limited sections of the RV endocardial surface to extrapolate global function, which may subject these measures to inaccuracies. See Table 1 for an overview of advantages and disadvantages of each 2D parameter.

**Table 1:** Advantages and limitations of one- and two-dimensional analysis techniques for right ventricular functional assessment.

	Definition	Advantages	Limitations
Doppler-derived tricuspid lateral annular systolic velocity (S’)	Assessment of the longitudinal excursion velocity of the lateral tricuspid annulus	• Reproducible • Easy to perform • Correlated with radionuclide angiography for functional discrimination • Validated in population-based studies • Does not depend on 2D image quality • Minimal required post-processing	• Assumes regional function is representative of the entire chamber • Angle dependent • Lack of normative data across sex and age
Tricuspid annular systolic excursion (TAPSE)	Measurement of longitudinal displacement of the tricuspid annulus in systole	• Reproducible • Reduced dependence on image quality • Correlated with Simpson's biplane right ventricular ejection fraction • Easy to perform • Minimal required post-processing	• Assumes regional function is representative of the entire chamber • Angle dependent • Varies with loading conditions
Free-wall and global longitudinal strain (FWS & 4CHLS respectively)	Percentage of myocardial shortening in the longitudinal plane	• Accounts for several RV segments • Correlated with CMR assessments • Feasible despite abnormal RV geometry • Load independent	• Angle dependent • High degree of variability across platforms • Requires post-processing with limited accessibility • Lack of normative data • Poor signal-to-noise ratio • Excludes RV outflow tract
Fractional area change (FAC)	Percentage difference between end systolic and end diastolic areas divided by end diastolic area	• Correlated with CMR assessments • Prognostic for heart failure, stroke, and death	• Excludes outflow tract and most of the RV body • High inter-observer variability • Varies with loading conditions • Tedious and time-consuming
Myocardial performance index (MPI)	Summation of RV iso-volumetric contraction and relaxation times divided by the RV ejection time	• Accounts for systolic and diastolic function • Well validated in healthy patients • Feasible despite abnormal RV geometry • Reduced dependence on image quality	• Reduced accuracy in the setting of tachycardia, irregular heart rhythms, and elevated RA pressures

## 3D echocardiography of the right heart

### Acquisition and analysis of the right ventricle

**Figure 4:**
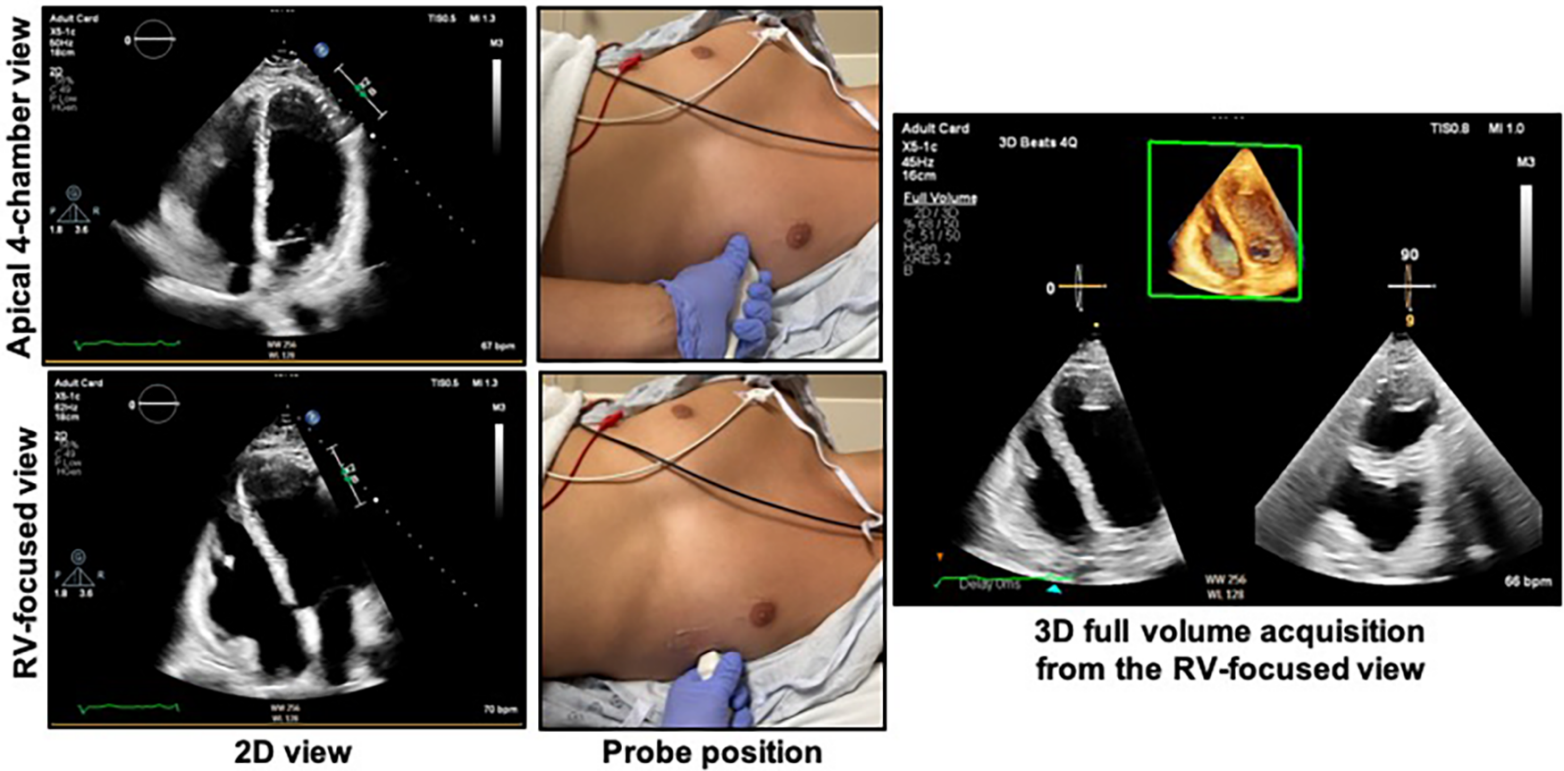
Obtaining the right ventricular focused view. The patient should be in the left lateral decubitus position with the left mid-clavicular, fifth intercostal region (approximate apex of the heart) positioned over the cut-out area of the bed if available. The top right shows the apical 4-chamber view as acquired from the probe position at top middle. The bottom left shows the RV-focused view as acquired from a more lateral location as shown in the bottom middle panel. The 3D dataset should be acquired from the RV-focused view to maximize capture of the RV free wall. See Figure 5 for the characteristics of an optimal 3D dataset. RV, right ventricle.

**Figure 5:**
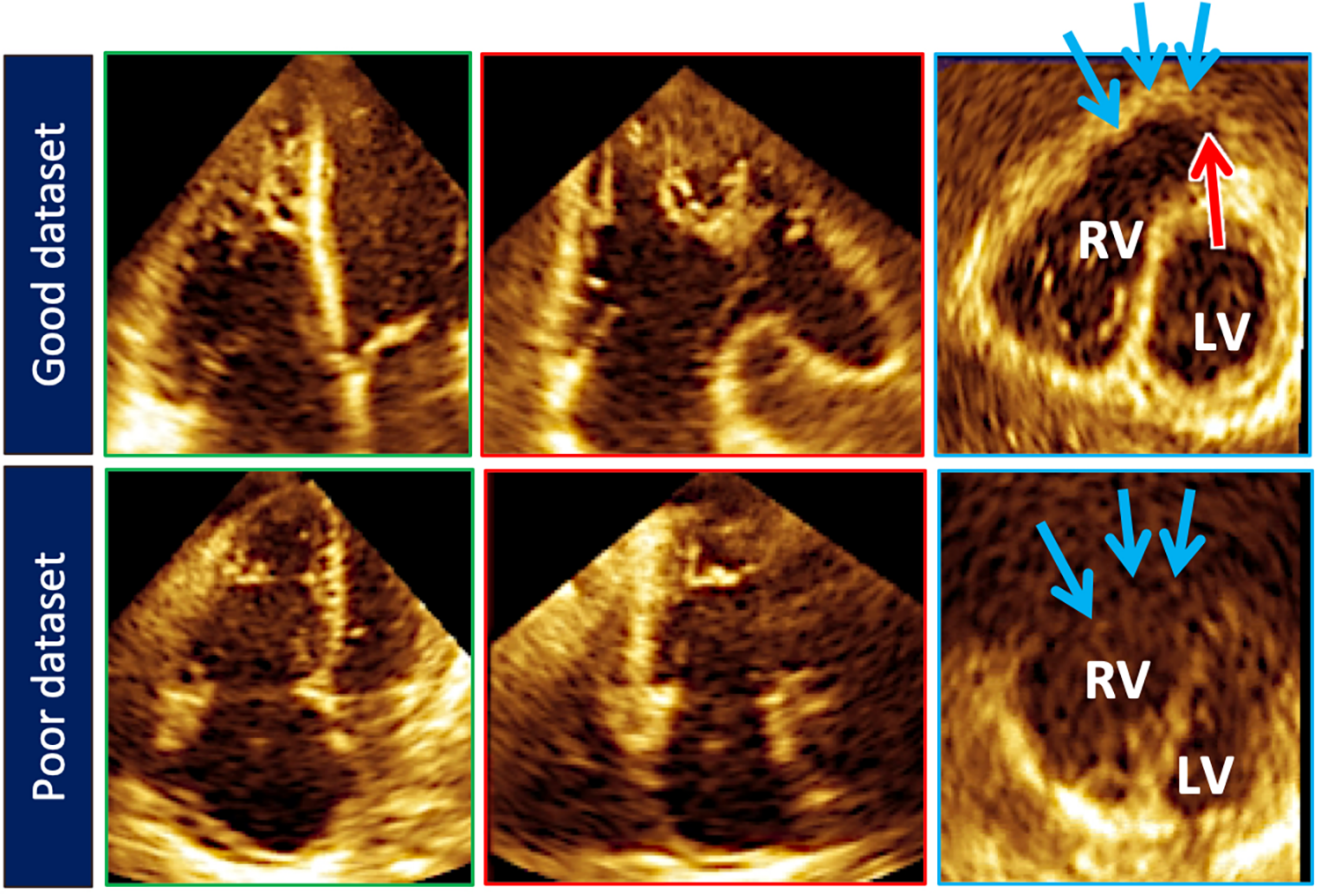
Top row illustrates cut planes through the optimal 3D right ventricular dataset. Note that the free-wall and right ventricular outflow tract borders (blue arrows) are well demarcated. Sometimes the pulmonary valve (red arrow) can be seen). Bottom row depicts a less than optimal 3D right ventricular dataset. The right ventricular outflow tract borders (blue arrows) are poorly seen.

**Figure 6:**
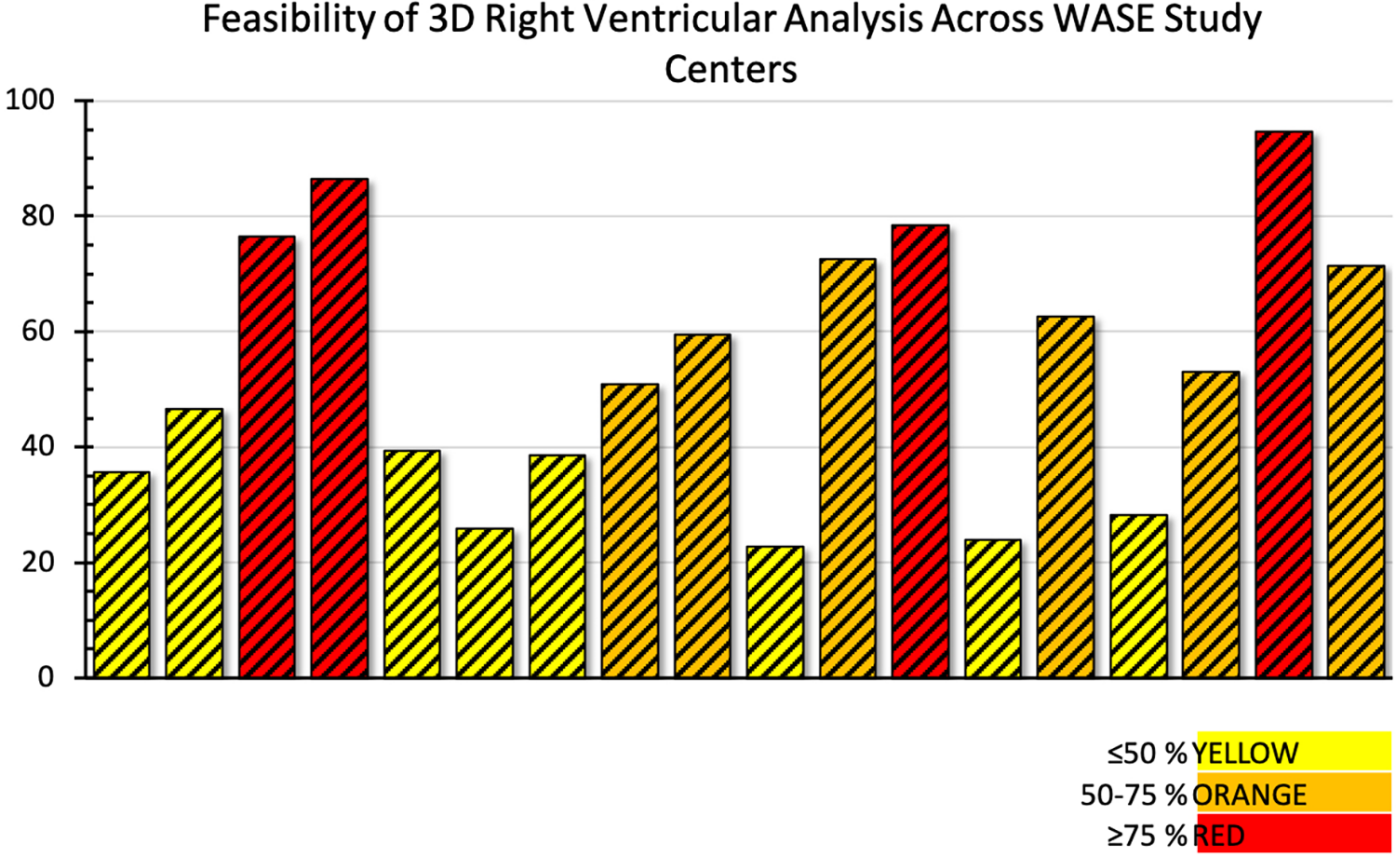
Feasibility of adequate 3D right ventricular dataset acquisition around the world. Data from the World Alliance Society of Echocardiography (WASE) study. Each bar represents one of the centers enrolled in the WASE study.

Acquisition of the 3D RV pyramidal dataset can be achieved using either a single or multi-beat approach from the RV-focused apical 4-chamber view (Figure 4). Notably, RV volumes and ejection fractions derived from 3D datasets acquired in the apical four-chamber view strongly correlate with measurements from the RV-focused view providing that the entire RV can be captured within the 3D dateset (12). Four to six beat acquisitions allow for higher temporal and spatial resolution enabling more optimal identification of end-diastolic and end-systolic phases for volumetric calculations (2). Prior validation studies with CMR in both children and adults have demonstrated sufficient frame rates ranging between 20 and 50 volumes per second to ensure reliable identification of cardiac timing (13, 14). Moreover, adequate patient cooperation with breath holding is critical to reduce stitch and dropout artifacts (2). Various clinical factors can adversely impact 3DE data collection including irregular cardiac rhythms, morbid obesity, inability to breath-hold, marked structurally abnormal RVs, mechanical ventilation, and mechanical support devices. In spite of these limitations on acoustic windows, transthoracic 3DE has exhibited exceptional feasibility in several large highly experienced cohorts ranging from 75% to 85% (15–17). The addition of ultrasound-enhancing agents has been shown to further augment performance with respect to reproducibility and correlation with CMR (18). In the World Alliance Society of Echocardiography (WASE) study, a worldwide cohort of centers with variable experience with 3D RV acquisition and analysis, feasibility of 3D RV acquisition dropped to 50%–60% with incomplete RV capture, typically anterior wall or apical drop-out being some of the main reasons for unanalyzable 3D RV data (Figure 5) (19). The ability to adequately capture the RV for 3D analysis is highly dependent on individual expertise to both acquire and analyze the 3D dataset. This expertise can vary widely from center to center as shown in the WASE study (Figure 6). According to this graph, the feasibility for RV analysis ranged anywhere from 20% to 95% and was dependent on the center in which the data was acquired, suggesting that it is possible to attain a level of expertise such that >80% of captured 3D RV data can be analyzed in patients with adequate 2D images. The major advantage of using 3D RV datasets for size and functional assessment is that this parameter represents the first echocardiography-based global method for RV functional assessment. Current 3D analysis software employs a volumetric approach to compute the total quantity of pixels within the RV endocardial surface in systole and diastole to obtain the respective volumes. This technique removes geometric assumptions and minimizes variability due to acquisition. Accordingly, volumes obtained from 3DE have demonstrated incremental improvements in accuracy and reproducibility compared to 2DE although they still underestimate volumes in comparison to CMR (5, 20). Fully automated methods of volumetric quantification based on machine-learning algorithms have been explored showing accurate, reproducible measurements following minimal revision (21).

The volumetric dataset provides multiple opportunities for post-processing analysis. Particular regions of the wide-angle acquisition can be visualized and interrogated for wall motion abnormalities, hypertrophy, or masses (3). 3D RV datasets enable better characterization of the complex contraction pattern of the RV including alterations caused by various pathologies. One increasingly popular method of 3D RV functional analysis involves the decomposition of the RV ejection fraction into longitudinal, radial, and anteroposterior components in order to investigate modifications in RV function with disease, which cannot be appreciated when studying its longitudinal performance alone. These contraction components have been investigated in patients with left ventricular dysfunction (22), pulmonary hypertension (23), and systemic right ventricles due to transposition of the great arteries (24) using the ReVISION software package (Argus Cognitive Inc., Lebanon, NH) (25, 26).

Conventional echocardiographic measures of RV function including 2D RV functional parameters (TAPSE, S', FAC, RV strain) and 3D RVEF (even if obtained using CMR) are highly load dependent and do not provide a clinically useful assessment of RV function in patients with secondary tricuspid regurgitation or significant pulmonary hypertension. Recent data on outcomes have identified measures of right ventricle-to-pulmonary artery coupling, which better estimate the impact of loading conditions on the RV, as important prognostic markers in these patients. In a recent study, RV volumes from 3D echocardiography were used to compute a surrogate of right ventricle-to-pulmonary artery coupling using the formula total RV forward stroke volume/end-systolic volume. This measure, when applied to patients with more than moderate tricuspid regurgitation, successfully predicted outcomes (including all cause death and hospitalization for heart failure) with better accuracy than RV ejection fraction and other measures of right-ventricle-to-pulmonary artery coupling using combinations of 2D and Doppler parameters raising the possibility that this marker could have a role in the assessment of RV function in patients undergoing percutaneous procedures for the tricuspid valve (27).

### Normal reference values for 3D right ventricular size and function parameters

Since its inception, numerous studies have sought to establish reference values for 3D chamber volumes and EF. Initial efforts displayed heterogenous findings, which could be attributed to inconsistencies in frame rates, volumetric analysis algorithms, and 3D imaging technology (28, 29). Recently, a large, multicenter investigation of 507 healthy volunteers evenly distributed across age and sex (17) showed for the first time, using 3DE, that men had larger right ventricular end-diastolic and end-systolic volumes compared to women even after indexation to body surface area, and that aging correlated with a consistent decline in volumes by decade. These results parallel those obtained from CMR in large populations of normal subjects (30, 31). From these findings, normative equations with allometric scaling were derived to assist with recognition of abnormal values. Even more recently, the World Alliance Society of Echocardiography (WASE) study also published normal values for 3D RV size and function parameters on 1,051 healthy volunteers, adding to the repertoire of 3D RV normal values with the added distinction of being the first 3D normal values study on a worldwide multi-ethnic cohort (19) (Table 2).

**Table 2:** Normal three-dimensional right ventricular and right atrial echocardiographic reference values.

	Source	N	Age (mean ± SD or range)	FR (Vol/s)	Analysis method	Tracing	Male (mean ± SD)										Female (mean ± SD)			
							N	EDVi ml/m^2^ (mean ± SD)	EDVi + 2SD ml/m^2^ upper limit	ESVi ml/m^2^ (mean ± SD)	ESVi + 2SD ml/m^2^ upper limit	EF % (mean ± SD)	EF % lower limit	N	EDVi ml/m^2^	EDVi + 2SD ml/m^2^ upper limit	ESVi ml/m^2^	ESVi + 2SD ml/m^2^ upper limit	EF % (mean ± SD)	EF % lower limit
RV	Gopal et al. (28)	71	56 ± 14	15–18	Short-axis disk summation	Manual	36	75 ± 13	101	38 ± 7	52	49 ± 10	29	37	65 ± 13	91	29 ± 11	51	56 ± 9	38
	Aune et al. (29)	166	29–80	NA	Automated border-detection (Qlab version 6)	Semi-automated	79	42 ± 11	64	17 ± 6	29	60 ± 11	38	87	38 ± 10	58	15 ± 5	25	62 ± 10	42
	Maffessanti et al. (17)	507	46 ± 16	26–40	Automated border-detection (TomTec version 1.2)	Semi-automated	247	59 ± 15	89	24 ± 9	42	60 ± 9	42	260	50 ± 11	72	19 ± 7	33	64 ± 9	46
	Addetia et al. (19)	1,051	47 ± 17	>15	Automated border-detection (TomTec)	Semi-automated	540	82 ± 21	124	37 ± 11	59	55 ± 5	45	511	70 ± 17	104	31 ± 9	49	57 ± 6	45
RA	Peluso et al. (37)	200	43 ± 15	NA	Automated border-detection (TomTec)	Semi-automated	87	12 ± 4	20	31 ± 8	47	61 ± 8	45	113	9 ± 3	15	27 ± 6	39	65 ± 8	49
	Soulat-Dufour et al. (41)	2,008	47 ± 17	NA	Automated border-detection (TomTec)	Semi-automated	1,033	11 ± 4	19	23 ± 7	37	53 ± 7	39	975	10 ± 3	16	21 ± 6	33	53 ± 7	39

Upper limits for volumes and lower limits for Ejection fraction also reported.

EDVi, indexed end-diastolic volume; ESVi, indexed end-systolic volume; EF, ejection fraction; FR, frame rate.

## 3D tricuspid annulus imaging and analysis

**Figure 7:**
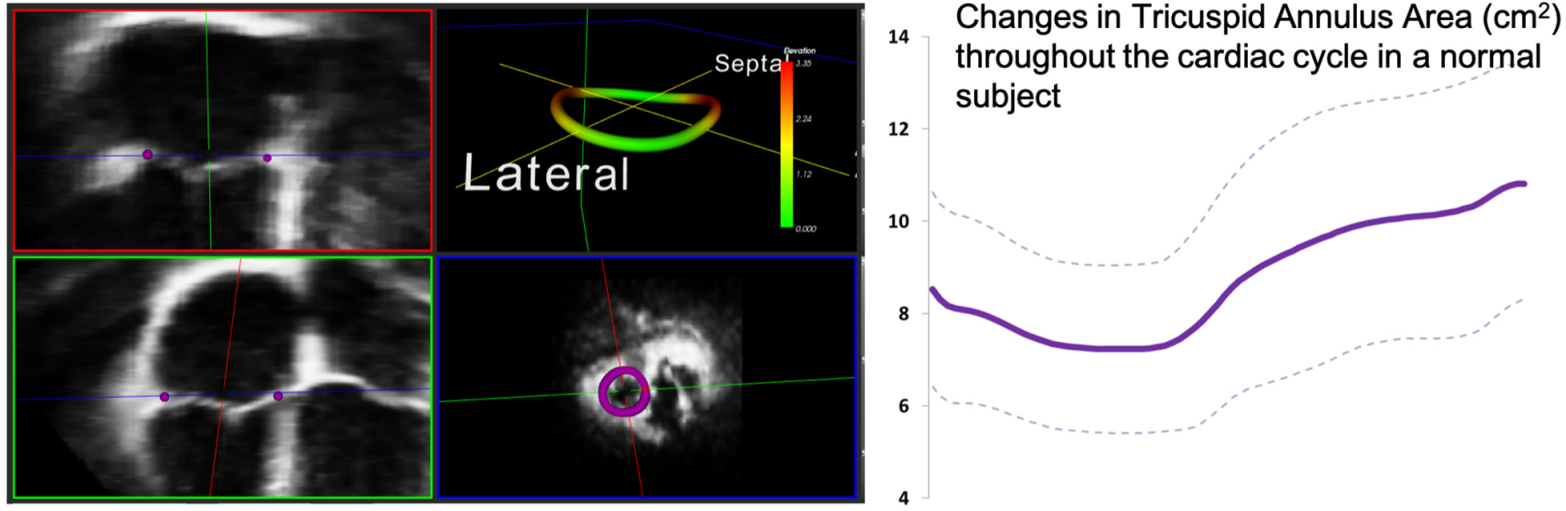
One example of customized software used to measure the 3D tricuspid annulus. Purple dots represent the initialization points from which the 3D annulus depiction (left panel, top right) is derived. The annulus is non-planar and therefore better assessed using 3D echocardiography. Dynamic analysis of the annulus allows calculation of changes in area and other parameters throughout the cardiac cycle (see graph right). Images courtesy of Federico Veronesi, PhD.

The tricuspid annulus (TA) forms the junction between the right atrium and the right ventricle. Its complex anatomy and dynamic behavior preclude systematic characterization with 2DE. Current guidelines recommend that the TA be measured in the apical 4-chamber view on transthoracic echocardiography. TA size and dynamics, however, are much more complicated (32). With 3DE, comprehensive static and dynamic assessment of the TA is possible (33). Visualization of the TA with 3DE begins with optimization of the RV-focused apical view. Narrow-angle or full volume acquisition from this plane adequately captures the TA. Accurate measurement of TA size and function even with current multi-planar reconstruction techniques is difficult due to the nonplanarity of the annulus necessitating manual or automated initialization of the leaflet hinge points with automated interpretation throughout the cardiac cycle (33, 34). Various programs are in existence or in development to assist with this step. One commercially available 3DE software package dedicated to the tricuspid valve was validated and utilized to develop sex-specific reference ranges for the TA. Importantly, TA sizes were shown to be underestimated by 2DE (35). See Figure 7 for results from an example software package.

## 3D of the right atrium

Right atrial (RA) assessment is of paramount importance in patients with diseases affecting the RV including pulmonary hypertension, heart failure (both reduced and preserved etiologies), and tricuspid regurgitation. The RA has been heralded as both “first chamber to live and the last to die” (36). With 3DE, it is possible to assess RA volumes, phasic function, and even remodelling (36, 37). In one study, increasing 3D RA sphericity index was found to be associated with clinical deterioration in patients with pulmonary arterial hypertension (36). Similar to the left atrium, RA physiology can be divided into 3 parts: (1) the reservoir phase, which corresponds to tricuspid valve closure and ventricular systole; (2) the conduit phase which corresponds to tricuspid valve opening and early ventricular diastole, and (3) the contractile or booster phase which reflects right atrial contraction (38). 3D RA volumes have been shown to be larger than the corresponding 2D volumes (37, 39, 40). Normal vales for 3D RA volumes are summarized in Table 2 (41).

## Diagnostic value of 3D echocardiography of the right ventricle

### Characterization of tricuspid valve disease

**Figure 8:**
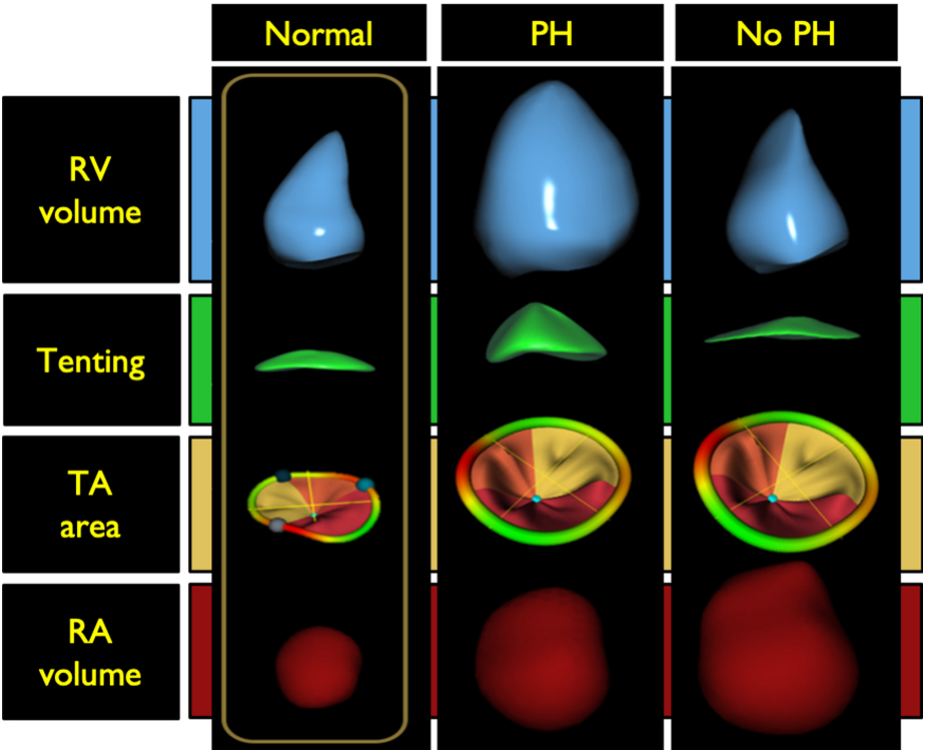
Right ventricular morphology changes associated with functional tricuspid regurgitation with and without pulmonary hypertension compared with a normal right ventricle (left panel). Middle panel illustrates the spherical right ventricular remodeling seen in patients with tricuspid regurgitation and pulmonary hypertension. In these patients there is often tricuspid valve leaflet tethering with some right atrial dilatation. The tricuspid annulus is typically minimally dilated and may in some cases be normal in size. The right panel illustrates ventricular remodeling in patients without pulmonary hypertension. This type of remodeling is typically seen in older adults with atrial fibrillation. There is notable right atrial and tricuspid annulus dilatation without leaflet tethering. Tricuspid annular dilation results in a diminished leaflet coaptation surface and increased rates of leaflet malcoaptation. In these patients, the RV dilates at the base, a phenomenon known as conical remodeling. PH, pulmonary hypertension; RV, right ventricle; TA, tricuspid annulus; RA right atrium. (Modified from Muraru D et al. Eur Heart J Cardiovasc Imaging. (2021) May 10;22(6):660−669).

Etiology of tricuspid regurgitation (TR) is closely coupled with right atrial and RV remodeling. Secondary or functional TR represents greater than 80% of TR in clinical practice and can be associated with or without pulmonary hypertension (PH) (42). These two TR categories have distinctive RV remodeling patterns which can be characterized using 3DE. Increases in RV afterload due to elevated pulmonary pressures can lead to spherical RV remodeling, papillary muscle displacement, tricuspid valve leaflet tethering, diminished leaflet coaptation surface, and tricuspid valve incompetence. The TA in these patients is often minimally dilated and may in some cases be normal in size. Flattening of the interventricular septum, a finding often seen in significant PH, likely also contributes to distortion of the tricuspid apparatus and TR. RV eccentricity index, quantified as the ratio between the long and perpendicular short-axis lengths at the mid- ventricular level in short-axis view has been shown to predict TR severity with high accuracy (43). Functional TR with PH has also been called ventricular functional TR, because the morphological changes in the right heart which sustain TR are seen mostly in the RV (Figure 8) (44, 45).

Functional TR without PH is typically seen in older adults with a high incidence of associated atrial fibrillation resulting in RA dilatation with subsequent TA dilatation without leaflet tethering (Figure 8). TA dilation results in a diminished leaflet coaptation surface and increased rate of leaflet malcoaptation. In these patients, the RV maintains its normal length but dilates at the base loosely labeled as conical remodeling (42, 44–46). Restoration of sinus rhythm has been shown to improve the degree of TR in some of these patients (47).

Detailed analysis of functional and morphologic changes associated with RV remodeling with 3DE can help to differentiate between the different causes of functional TR and the impact on the RV (44–46). Regional quantitative curvature analysis has previously been implemented to characterize alterations in RV shape attributed to PH using a quantitative approach, demonstrating bulging of the interventricular septum into the left ventricle and greater convexity of the RV free wall throughout the cardiac cycle compared to normal controls. This bulging phenomenon is also known as the “D-shaped left ventricle” or “D-sign”, a finding that can also be appreciated on the 2D parasternal short-axis view (Figure 8) (48).

### Implantable device lead complications

**Figure 9:**
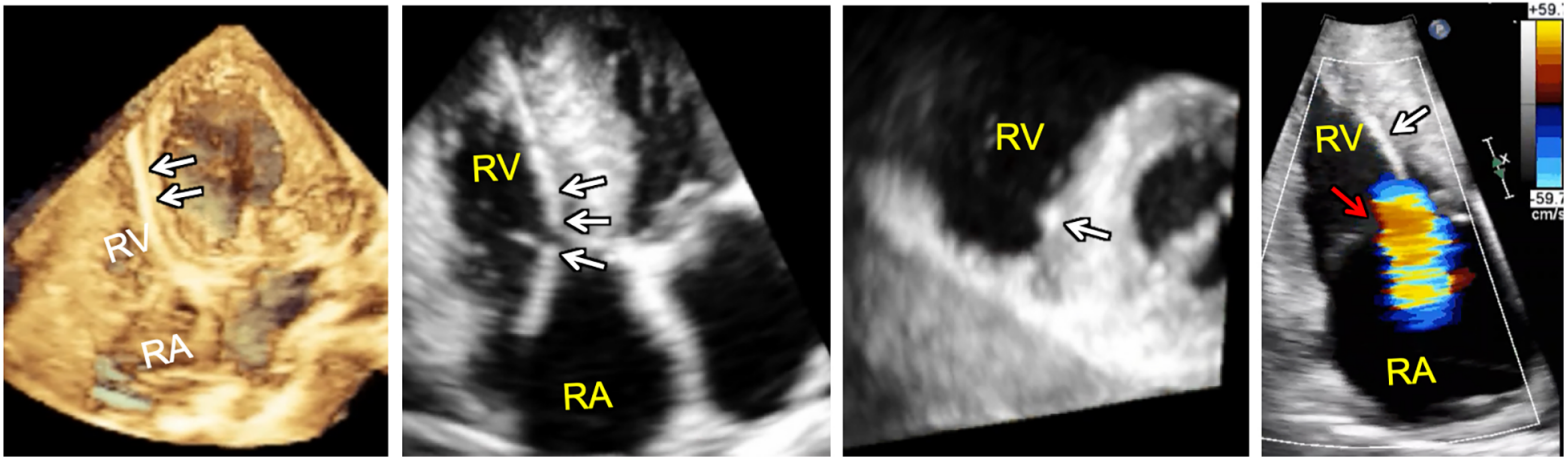
3D full volume dataset of the right ventricle (far left) and adjacent cut planes through the 3D dataset (left, middle and right, middle) showing device-lead (white arrows) impinging on the septal leaflet and sub-tricuspid apparatus in the region of the right ventricular septum resulting in tricuspid regurgitation (far right). Note that the tricuspid leaflet coaptation zone (red arrow) is not the origin of tricuspid regurgitation in this case. In fact, tricuspid regurgitation is originating at the point of device-lead contact with the septal leaflet.

Device-lead related interference with the tricuspid valve apparatus is a potential complication that is incompletely described by 2DE as the device-lead is really only seen in its entirety in less than 20% of cases (49). The addition of 3D RV imaging has improved non-invasive assessment of the relationship between the tricuspid apparatus and device leads enabling direct visualization of the both the TV leaflets and the sub-tricuspid apparatus making it possible to determine the presence or absence of interference in select cases (50–52). According to one study, leads demonstrating leaflet impingement on 3DE were associated with a greater degree of TR with a median vena contracta of 0.62 cm compared to 0.27 cm in patients without 3DE evidence of lead-related leaflet interference. Importantly, positioning of the lead in the commissure resulted in less frequent interaction, suggesting that echocardiographic guidance of or follow-up after lead placement may be beneficial in some cases (52). More recent data suggests that lead-related tricuspid valve interference can be associated with the tricuspid valve leaflet(s) alone, the sub-tricuspid apparatus alone, or both the leaflet(s) and the sub-tricuspid apparatus (Figure 9) (53).

Given the increasing interest in percutaneous tricuspid valve repair in patients with heart failure, it is likely that there will be a parallel need to better diagnose lead-related interference with the tricuspid valve apparatus as any associated interference may impact the success of any procedure chosen to repair the tricuspid valve. 3DE shows promise in this area of diagnosis.

### Arrhythmogenic right ventricular cardiomyopathy (ARVC)

**Figure 10:**
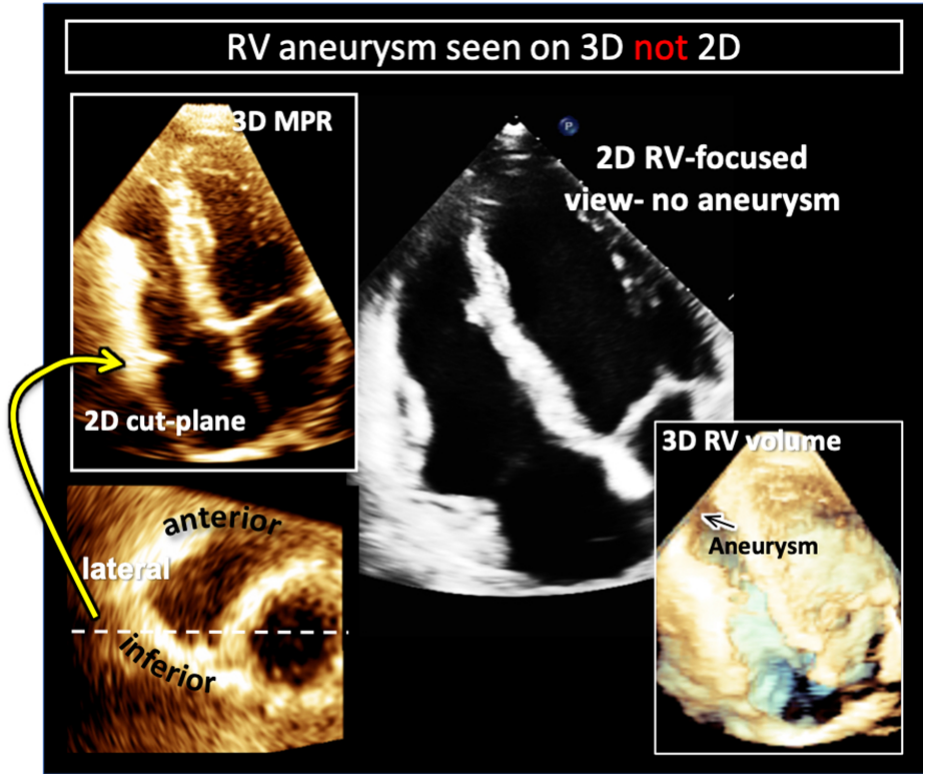
Example of right ventricular free-wall aneurysm detection with 3D (left) compared to 2DE in a patient with arrhythmogenic right ventricular cardiomyopathy (ARVC). Two-dimensional cut-planes from a full-volume 3D dataset enables visualization of the apical aneurysm (left) with targeted interrogation of the right ventricular free-wall (left, top) on short-axis imaging planes (left bottom). Here the aneurysm is noted to be on the infero-lateral free wall (white dotted line and yellow arrow). In the same patient the aneurysm is not visualized in the 2D right-ventricular focused view (right, top). Cropping into the 3D full volume also allows visualization of the apical aneurysm (black arrow on right, bottom).

Echocardiography represents a common first-line imaging modality for both diagnosis and follow-up of ARVC (54). Although CMR is considered the gold standard for meeting the imaging criteria for diagnosis, recent investigations have demonstrated a high concordance in diagnostic performance between 3DE and CMR when either is combined with 2DE. Moreover, 3DE enhances the ability to diagnose wall motion abnormalities and aneurysms which are critical to meeting imaging criteria for the diagnosis of the disease (Figure 10) (55, 56). 3DE specifically outperformed 2DE in detection of wall motion abnormalities and exhibited comparable detection rates to CMR (56). These suggest the possibility of 3D echocardiography-guided diagnosis and follow-up of these patients especially in those instances where CMR is not easy to obtain.

### Congenital heart disease

The asymmetrical remodeling of the RV often observed in patients with congenital heart disease can limit the efficacy of conventional 2D size and function assessment of the RV due to inaccurate geometric assumptions. These geometric assumptions may be overcome by 3DE. Volumes and ejection fractions acquired from 3DE datasets have been shown to correlate well with CMR measurements in addition to demonstrating lower interobserver variability than corresponding measurements obtained on 2DE (57, 58). However, in conditions with progressive RV enlargement including repaired tetralogy of Fallot (TOF) and transposition of the great arteries 3DE has been shown to systematically underestimate volumes while overestimating RVEF (59, 60). These studies were notably performed using earlier software versions that may impact their current applicability. More recent versions of the 3D RV analysis software are easier to use and provide short-axis cut-planes for tracing volumes. A recent investigation in patients with systemic RVs, revealed alterations in contractile mechanisms between patients with transposition of the great arteries (TGA) and congenitally corrected TGA, with motion along the anteroposterior plane dominating RV contraction in TGA and while all components (anteroposterior, longitudinal and radial) contribute equally to ventricular ejection fraction in congenitally corrected TGA (24, 61). 3DE has also facilitated characterization of RV shape and strain changes in conditions that result in chronic pressure and/or volume overload. Patients with TOF exhibited less curvature of the free wall, a more convex intraventricular septum, and significantly impaired RV strain compared to controls (62, 63).

## Prognostic value of 3D echocardiography of the RV

### Mixed population studies

RV function is reported to have prognostic significance in a variety of cardiovascular diseases including heart failure, PH, and coronary artery disease (Table 3) (1, 64–70). Importantly, 2D RV functional assessment can sometimes be inaccurate when used in these disease states. In post-cardiac surgery patients, for instance, due in part to geometrical changes associated with RV protection during bypass or alterations in interventricular septal motion after surgery, the longitudinal excursion of the RV is typically reduced despite preservation of overall RV function (71–74). TAPSE and RV S', therefore, underestimate RV function in this population and cannot be used to assess RV function. This phenomenon extends to at least 1 year post operatively. Global assessment of RV function with 3DE, however, can more accurately measure RV function in these patients suggesting that 3D RV assessment likely has wider applicability than 2DE in the remodeled or abnormal RV (75). 3D RV size and function parameters have been shown to have incremental value in the prediction of outcomes independent of left ventricular ejection fraction (67). In one study, the prevalence of patients with systolic LV dysfunction (left ventricular ejection fraction <52%) increased with worsening 3D RV ejection fraction across a population of consecutive patients with various cardiac conditions who had 3D RV acquisitions performed (68). In another retrospective study of 446 patients over a median follow up of 4.1 years, the authors showed that 3D RV ejection fraction offered incremental value over clinical risk factors and other echocardiographic parameters, including left ventricular systolic and diastolic function, for predicting future adverse outcomes including cardiac death and major adverse cardiac events (MACE) (1). The 3D RV ejection fraction cut-off values to stratify worsened prognosis were 35% for cardiac death and 41% for MACE. A prospective study of 50 patients, with a median follow-up of 16 months, found that 3D RV ejection fraction remained the only independent predictor of MACE after controlling for both clinical and echocardiographic variables, including age, New York Heart Association class, E/e' ratio, and left atrial volume index. By ROC analysis, the optimal RV ejection fraction cut-off value for event prediction was 43.4% (AUC = 0.77, *p* = 0.001), and RV ejection fraction remained an independent predictor in multivariable models when treated as a categorical variable using the cut-off of 43.4%. Categorization of 3D RV ejection fraction into the following partition values: 45%, 40%, and 30% (i.e., very low risk for mortality (RV ejection fraction >45%), low risk (40%< RV ejection fraction ≤45%), moderate risk (30%< RV ejection fraction ≤40%), and high risk (RV ejection fraction ≤30%), stratified the population into high, moderate, and low risk of cardiac death and MACE (68). Furthermore, a recent meta-analysis of ten studies including 1,928 patients identified a robust association between a one standard-deviation reduction in 3D RV ejection fraction and adverse outcomes that was stronger than 2D measures including TAPSE, FAC, and FWS (6).

**Table 3:** Studies using RV volumes, EF and strain as prognostic indices.

Publication	Study aim (s)	Parameters studied	Population and methods	Prognostic parameters
Li et al. (86)	To predict adverse clinical outcomes in CTEPH patients with 3D RV indices	RV volumes and EF	• 151 consecutive CTEPH pts Median follow up: 19.7 months	3D analysis of RVEF was a predictor of adverse clinical outcomes [hazard ratio, 1.576; 95% confidence interval (CI), 1.046–2.372; *P* = 0.030]
Meng et al. (69)	To determine whether 3D-STE parameters were the more powerful predictors of poor outcomes in HFpEF patients compared with 2D-STE indices	3D RV volumes, EF and 3D-RVFWS	• 81 consecutive patients with HFpEF After a median follow-up period of 17 months, 39 (48%) patients reached the end point of cardiovascular events	3D-STE parameters are powerful predictors of poor outcomes, providing a similar predictive value as 2D-STE indices in patients with HFpEF
Vîjîiac et al. (66)	To evaluate role of 2D RV strain and 3D RVEF in predicting adverse outcome in patients with non-ischemic dilated cardiomyopathy.	RV global longitudinal strain, RV FWS, 3DRVEF	• 50 eligible patients Median follow-up of 16 months, 29 patients reached the primary endpoint	3D RVEF is an independent predictor of major adverse cardiovascular events in patients with dilated cardiomyopathy
Wang et al. (103)	To investigate whether 2D strain and 3DE could identify impaired RV function after anthracycline exposure	RV 4CHLS, RV FWS, 3D RVEF	61 patients with diffuse large B-cell lymphoma treated with anthracycline were studied	2D STE and 3D echocardiography are valuable methods for evaluating anthracycline-related impairment of RV function in DLBCL patients receiving chemotherapy. RV FWLS and RVEF are reliable predictors of RV systolic dysfunction
Liu et al. (104)	To explore the value of RV parameters detected by 3DE in risk stratification in PAH patients	RV volumes, EF, RV FWS	91 PAH patients (34 ± 12 years, 25 males) were enrolled, among which, 42 were classified into low-risk group, while 49 were intermediate-high risk group	RV volumes, EF and free wall strain detected by 3DE were independent predictors of intermediate-high risk stratification in PAH patients, among which, RVEF showed the best predictive capacity
Tokodi et al. (73)	To explore the association between RV contraction patterns pre mitral valve surgery and post-operative RV dysfunction	3D RVEF, radial and longitudinal components of function	• 42 patients (63 ± 11 years) undergoing MV surgery Patients had pre-operative, at-discharge, and 6-months post-operative TTE's	There was a shift in RV contraction mechanics from longitudinal contraction predominance pre- and radial pre-dominance in the first 6 months post MV surgery. Pre-operative LVEF predicted post-operative RV dysfunction in patients undergoing MV surgery
Nagata et al. (1)	To determine whether 3DRVEF predicts future cardiovascular events	RV volumes, EF	446 patients with various cardiovascular diseases	3D TTE-determined RV EF was independently associated with cardiac outcomes. 3D RVEF offered incremental value over clinical risk factors and other echocardiographic parameters including LV systolic and diastolic function for predicting adverse outcome
Tamborini et al. (105)	To assess RA, RV and TA geometry and function in patients undergoing MV repair +/− TV annuloplasty	3D RA, RV volumes and tricuspid annulus	103 patients undergoing MV surgery without (54 cases) or with (49 cases) concomitant TV annuloplasty and 40 healthy controls	Patients undergoing MV surgery and TV annuloplasty had an increased TA dimensions and a more advanced remodelling of right heart chambers reflecting more advanced disease
Vitarelli et al. (70)	To investigate whether 2D, 3D RV assessment could result in better correlation with hemodynamic variables indicative of heart failure	2D and 3D volumes, EF and strain	• 73 patients (53 ± 13 years; 44% male) with chronic PH of different etiologies were studied by cardiac catheterization and echocardiography 25 precapillary PH, 23 obstructive pulmonary heart disease, 23 postcapillary PH from mitral regurgitation and 30 healthy controls	ROC curves: detecting hemodynamic signs of RV failure were 39% for 3D-RVEF (AUC 0.89), −17% for 3DGFW-RVLS (AUC 0.88), −18% for GFW-RVLS (AUC 0.88), −16% for apical-free-wall longitudinal strain (AUC 0.85), 16 mm for TAPSE (AUC 0.67), and 38% for RV-FAC (AUC 0.62)

3D, 3-dimensional; CTEPH, chronic thromboembolic pulmonary hypertension; DLBCL, doxorubicin chemotherapy; HFpEF, heart failure with preserved ejection fraction; EF, ejection fraction; MV, mitral valve; RA, right atrial; RV, right ventricular; RVFWS, right ventricular free wall strain; STE, strain echocardiography; TA, tricuspid annulus; TV, tricuspid valve; TTE, transthoracic echocardiography; PAH, pulmonary arterial hypertension.

3D RV parameters have also been shown to have utility in predicting adverse events in patients with heart failure with preserved ejection fraction. Meng et al. found that lower RV ejection fraction and 3D RV longitudinal strain of free wall were associated with heart failure, hospitalization, or death (69).

### Pulmonary hypertension

Pulmonary hypertension has garnered a great deal of interest in the study of 3D RV applications, because the entire spectrum of RV remodelling can be documented in this cohort of patients (76). Together with 3D volumes, 3D deformation indices have an important role in the prognosis of PH patients. Specifically, changes in RV function and 3D RV area strain have been shown to be of prognostic importance and correlate more strongly with hemodynamics in RV failure than conventional echo indices (77, 78). In the pediatric PH population, 3D volumes, 3D RV ejection fraction, FAC, and free wall RV longitudinal strain were significantly associated with outcome (79). The ratio of 3D RV stroke volume to end-systolic volume (ESV) ratio as an estimate of RV-arterial coupling correlated with RV strain and was found to be a strong predictor of adverse clinical events in pediatric patients with PH (80).

### Secondary tricuspid regurgitation

The ability of 3DE to characterize patterns of chamber remodelling resulting in secondary TR has prompted investigation into the prognostic impact of atrial and ventricular TR. In a population of patients with moderate-severe TR, patients with atrial TR comparably exhibited a lower rate of all-cause death and hospitalization due to heart failure (81). Categorization of TR severity as mild, moderate, and severe based on 3DE-derived regurgitant volume and effective regurgitant orifice area (82) revealed progressively higher rates of all-cause death and hospitalization due to heart failure with increasing TR severity, imparting the importance of the severity grading (81, 83). As aforementioned, estimation of right ventricular-to-pulmonary artery coupling in these patients can be challenging and therefore surrogate measures derived from 3DE have been investigated. In one study, a ratio between RV forward stroke volume and end-systolic volume less than 0.40 was associated with a higher risk of death and heart failure hospitalization (27).

It is important to note that structural tricuspid valve procedures and trials do not yet employ 3D indices of the right ventricle or tricuspid valve for decision-making (84). Data on the utility of 3D RV analysis in this space should become available shortly from the TRILUMINATE trial imaging sub-study. Additionally, there are two other studies incorporating 3D of the tricuspid valve which are currently recruiting patients: NCT05130775 and NCT05747404. The results of these studies are highly anticipated.

### Pulmonary embolism/chronic thromboembolic pulmonary hypertension

Understanding the impact of a pulmonary embolism on the RV is essential in determining severity and in assessing recovery in follow-up. Here, 3DE of the RV has been shown to have the potential to serve as a useful adjunctive tool in both the acute and chronic settings. In the case of acute sub-massive pulmonary embolism, a reduced 3D RV ejection fraction was noted to be the most sensitive predictor of adverse events and signified a longer time for recovery of function at follow-up compared to 2D parameters (85). In an adult population of patients with chronic thromboembolic disease, a machine learning-based calculation of RV ejection fraction from 3D RV datasets, with a determined cut-off of approximately 31%, was a significant predictor of adverse events these in patients (86). Multiple studies utilizing 3DE have sought to characterize RV function before and after pulmonary thromboendarterectomy for management of chronic thromboembolic pulmonary hypertension. Findings revealed a consistent trend of significantly reduced chamber volume and improved systolic performance post-operatively that persisted at long-term follow-up (87–89).

## Future directions

While, in the last decade, speckle tracking analysis of left ventricular performance has significantly fueled the investigation of LV mechanics and its prognostic value, the exploration of 3D RV strain has not advanced. The complex morphology of the RV chamber, the difficulty associated with imaging this chamber and the lack of dedicated software are all factors that have contributed to the sparse interest in 3D RV mechanics. More recently, with the development of different software solutions, characterization of 3D RV mechanics in healthy volunteers (90), and a variety of cardiac diseases (91) has been initiated by some investigators. 3D RV strain has been shown to be independently associated with short-term outcomes in patients undergoing cardiac surgery (73, 92), the severity of obstructive sleep apnea (93), prognostication in heart failure with preserved ejection fraction (69) and pulmonary hypertension (94, 95).

Studies performed on the left ventricle have indicated that its shape carries information independent from conventional functional measurements and is related to prognosis (96, 97). Alterations in 3D left ventricular shape have also been cited as an early manifestation of remodeling in patients with severe mitral regurgitation and normal left ventricular ejection fraction (98). Unlike the left ventricle, the peculiar morphology of the RV does not allow its shape to be simplified to resemble a simple geometrical model. For this reason, RV shape has largely been studied in terms of regional curvedness. Subtle changes in the physiological condition is reflected by local adaptation of the RV wall which can be quantitatively measured by its curvature, for example, a more locally convex or concave wall. RV curvature is altered as a consequence of the remodeling induced by pathological conditions, such as pulmonary hypertension (48, 99), volume overload (62), but also in the settings of congenital heart disease (63) and in patients with mechanical circulatory support (100).

**Figure 11:**
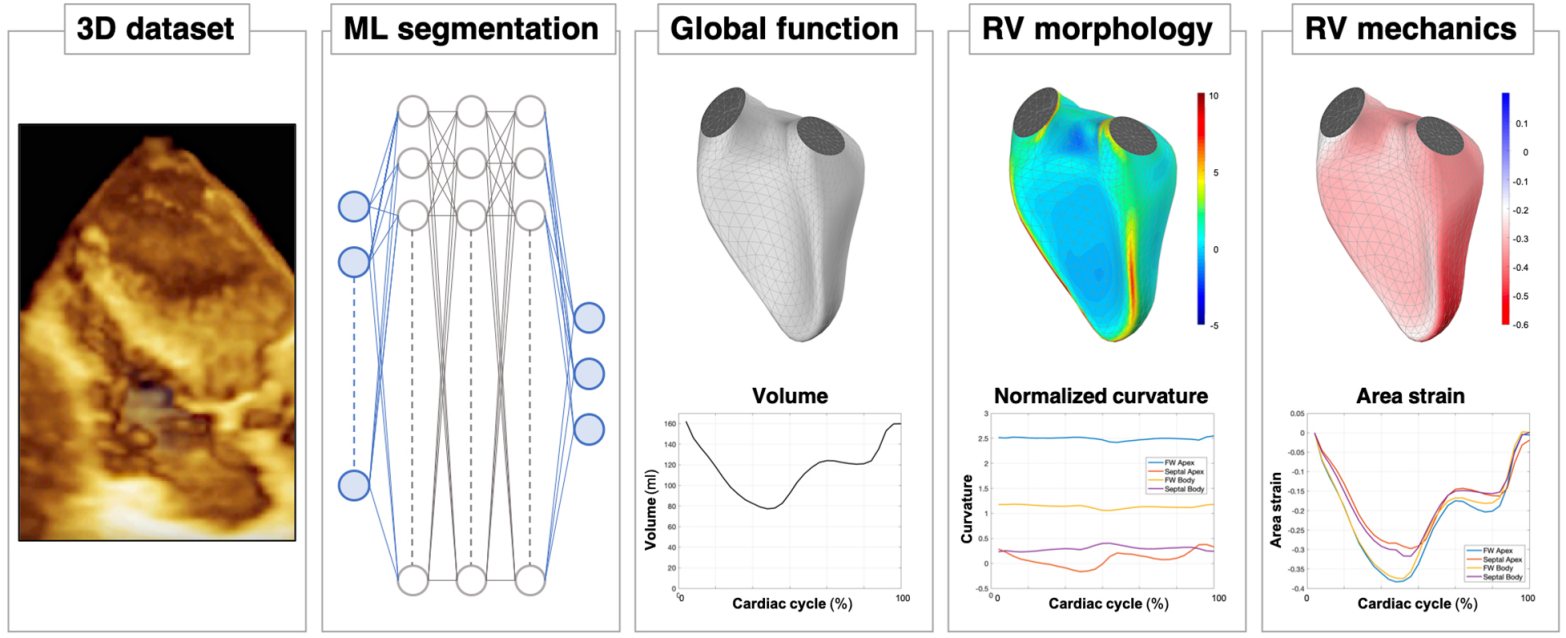
A look into the future of right ventricular assessment by 3D echocardiography: after the acquisition of the 3D dataset of the RV chamber, eventually supported by artificial intelligence (AI)-guided systems, a machine learning (ML) algorithm will be able to obtain a rapid, accurate and reproducible segmentation of the RV cavity. Based on these time-evolving surfaces, a comprehensive analysis of the RV will be automatically carried out, from conventional functional analysis (volumes and ejection fraction) to more sophisticated quantitative analyses, including morphological analysis in terms of local curvature or local mechanical function, as assessed by displacement and strain measurements.

While it has been established that 3D RV measurements are more accurate and prognostic than 2D parameters, it is notable that in the vast majority of centers around the world 3D RV dataset acquisition is not able to meet the requirements to perform a reliable quantitative analysis (19). Indeed, even when data is adequate, quantitative analysis is usually time consuming and requires well-trained echocardiographers to ensure adequate accuracy and reproducibility. This paradox produces a challenge for image-guided artificial intelligence systems of the future to predict, display, and guide echocardiographers and sonographers during image acquisition with the goal to increase the feasibility rate of subsequent 3D RV analysis. This ability already exists in a rudimentary form to guide novices to acquire and display diagnostic quality 2D images (101). Artificial intelligence solutions could also be developed with the potential to fully automate the identification and segmentation of the RV. Fusion of data obtained from different imaging planes/probe positions could theoretically be used to, for instance, address the problem of acquiring a complete dataset in abnormal and large RVs. Deep learning algorithms have already shown promise in accurately predicting 3D RV ejection fractions from two-dimensional images, identifying RV dysfunction with an accuracy equivalent to an expert (78%) and with the additional potential to predict major adverse cardiac events (102). If this combination of AI-guided algorithm allowing quality controlled acquisition, automated segmentation and multiparametric—functional, morphological and mechanical and even hemodynamic (if acquisition of invasive RV pressures and 3D echocardiographic datasets could be acquired simultaneously to develop pressure-volume loops)—quantitative analysis becomes available, it would become an important, accurate and reproducible tool for the assessment and understanding of RV pathophysiology (Figure 11).

## Conclusions

3D echocardiography offers substantial advantages for comprehensively evaluating the right ventricle compared to conventional 2D echocardiographic assessment. The growth of 3DE has corresponded with an increasing number of diagnostic applications requiring global chamber assessments as well as considerable investigation into the prognostic significance of 3DE measures. Novel analysis techniques including RV strain and shape combined with automated interpretations may further expand the role of 3DE in clinical practice.
